# Specific Denitrifying and Dissimilatory Nitrate Reduction to Ammonium Bacteria Assisted the Recovery of Anammox Community From Nitrite Inhibition

**DOI:** 10.3389/fmicb.2021.781156

**Published:** 2022-01-20

**Authors:** Xuejiao Qiao, Liyu Zhang, Zhiguang Qiu, Li Wang, Yang Wu, Chunfang Deng, Jia Su, Xue Zhang, Yuexing Wang, Bing Li, Lijie Zhou, Anthony Y. W. Ma, Wei-Qin Zhuang, Ke Yu

**Affiliations:** ^1^School of Environment and Energy, Peking University Shenzhen Graduate School, Shenzhen, China; ^2^Laboratory of Municipal Wastewater Treatment and Reutilization Engineering, Shenzhen Water Group, Shenzhen, China; ^3^Shenzhen Engineering Research Laboratory for Sludge and Food Waste Treatment and Resource Recovery, Tsinghua Shenzhen International Graduate School, Tsinghua University, Shenzhen, China; ^4^College of Chemistry and Environmental Engineering, Shenzhen University, Shenzhen, China; ^5^Green Living and Innovation Division, Hong Kong Productivity Council, Hong Kong, Hong Kong SAR, China; ^6^Department of Civil and Environmental Engineering, The University of Auckland, Auckland, New Zealand

**Keywords:** nitrite inhibition, anaerobic ammonium oxidation bacteria, dissimilatory nitrate reduction to ammonium bacteria, nitrogen metabolic pathways, denitrifying bacteria

## Abstract

The anaerobic ammonium oxidation (anammox) by autotrophic anaerobic ammonia-oxidizing bacteria (AnAOB) is a biological process used to remove reactive nitrogen from wastewater. It has been repeatedly reported that elevated nitrite concentrations can severely inhibit the growth of AnAOB, which renders the anammox process challenging for industrial-scale applications. Both denitrifying (DN) and dissimilatory nitrate reduction to ammonium (DNRA) bacteria can potentially consume excess nitrite in an anammox system to prevent its inhibitory effect on AnAOB. However, metabolic interactions among DN, DNRA, and AnAOB bacteria under elevated nitrite conditions remain to be elucidated at metabolic resolutions. In this study, a laboratory-scale anammox bioreactor was used to conduct an investigation of the microbial shift and functional interactions of AnAOB, DN, and DNRA bacteria during a long-term nitrite inhibition to eventual self-recovery episode. The relative abundance of AnAOB first decreased due to high nitrite concentration, which lowered the system’s nitrogen removal efficiency, but then recovered automatically without any external interference. Based on the relative abundance variations of genomes in the inhibition, adaptation, and recovery periods, we found that DN and DNRA bacteria could be divided into three niche groups: type I (types Ia and Ib) that includes mainly DN bacteria and type II and type III that include primarily DNRA bacteria. Type Ia and type II bacteria outcompeted other bacteria in the inhibition and adaptation periods, respectively. They were recognized as potential nitrite scavengers at high nitrite concentrations, contributing to stabilizing the nitrite concentration and the eventual recovery of the anammox system. These findings shed light on the potential engineering solutions to maintain a robust and efficient industrial-scale anammox process.

## Introduction

Anaerobic ammonium oxidization (anammox) has gained significant momentum as a highly efficient, cost-effective, and environment-friendly biological nitrogen removal process compared with the conventional nitrification–denitrification processes (Ismail et al., 2019). Anammox is mediated by the autotrophic anaerobic ammonia-oxidizing bacteria (AnAOB), which oxidize ammonium (NH_4_^+^) using nitrite (NO_2_^–^) as an electron acceptor under anaerobic conditions and produce nitrogen gas (N_2_) and nitrate (NO_3_^–^) (Bonassa et al., 2021). The anammox process has primarily been used to treat ammonium-rich wastewater (Alejandro et al., 2018), such as anaerobic digestion liquid of sludge, landfill leachate, urban domestic sewage, swine wastewater, or monosodium glutamate wastewater. Due to the extremely high ammonia nitrogen content (800–3,000 mg/L) in these types of wastewater, a preliminary step before the implementation of the anammox process is to oxidize approximately half of the ammonium into nitrite, known as partial nitritation, by ammonium oxidizing bacteria in the wastewater (Li et al., 2019). However, the nitritation process is difficult to control, often resulting in an overproduction of nitrite (Tao et al., 2012), leading to the inhibition of the anammox process.

This possible inhibitory effect makes controlling nitrite concentration a focus in implementing the anammox process. The inhibitory nitrite concentrations reported vary from 5 to 750 mg N L^–1^ (Zekker et al., 2017; Cho et al., 2020) in various anammox-based systems. Although the broad range of inhibitory nitrite concentrations can be attributed to variations in experimental conditions and operating modes (pH, temperature, experimental continuity, etc.), it implies that a mechanistic understanding of nitrite inhibition and nitrite resistance of the anammox microbial communities remains to be elucidated. Furthermore, due to the slow growth of AnAOB (Wang et al., 2016), the recovery from inhibition/death caused by prolonged high nitrite shock was proved to be difficult (van der Star et al., 2007) and puzzling even when a successful recovery did happen (Lotti et al., 2012).

Denitrifying (DN) and the dissimilatory nitrate reduction to ammonium (DNRA) bacteria can metabolize nitrite and potentially promote the recovery process of the anammox system from the nitrite shock. AnAOB is a key player in nitrogen cycling, whereas DN and DNRA bacteria are also important participants of the anammox system (Gonzalez-Gil et al., 2015; Keren et al., 2020). To date, the vast majority of identified AnAOB belongs to Planctomycetes, including six genera that have been reported so far, i.e., *Candidatus* (*Ca.*) Brocadia, *Ca.* Jettenia, *Ca.* Kuenenia, *Ca.* Scalindua, *Ca.* Anammoxoglobus, and *Ca.* Anammoxomicrobium (Ibrahim et al., 2016). DN and DNRA bacteria are mainly heterotrophs from a variety of phyla, for example, Proteobacteria, Chloroflexi, and Actinobacteria (Zhao et al., 2019; Keren et al., 2020). Many studies have pointed out that autotrophic organisms such as AnAOB can release soluble microbial products and extracellular polymeric substances. Environmental stimuli can cause the immediate release of these substances from the autotrophs (Ma et al., 2012; Chu et al., 2015; Zhang et al., 2016). Heterotrophic bacteria, including DN and DNRA bacteria, can thus live on these substances for survival and cater to important nitrogen cycling processes (Guo et al., 2016; Hou et al., 2017; Zhu et al., 2017). DN bacteria can reduce NO_3_^–^ and NO_2_^–^ to N_2_ (Zhou et al., 2014), whereas DNRA bacteria can reduce them to NH_4_^+^. The function of DNRA bacteria under high nitrite concentrations was not clear. DN bacteria from Proteobacteria, however, were reported to aid in reducing nitrite to nitrogen gas. In short, the nitrite denitrification genes in DN bacteria had higher abundance after the inhibition phase, and their cooperation could prevent the nitrite inhibition of anammox bacteria when the influent nitrite concentration was higher (Zhao et al., 2019). Thus, the DN/DNRA bacteria can potentially reduce the NO_2_^–^ concentration and then balance the NO_2_^–^/NH_4_^+^ ratio to create a preferable environment for the anammox process (Wang et al., 2018; Zhao et al., 2019). There were a number of studies using amplicon sequencing targeting 16S ribosomal RNA (rRNA) gene to decode the bacterial communities (Ren et al., 2014; Liang et al., 2015), whereas others have used shotgun metagenomic approaches to understand functional genomes in anammox systems (Guo et al., 2016; Ciesielski et al., 2018; Sun et al., 2018; Tang et al., 2018). Overall, the metagenomic analysis is more powerful in revealing microbial functions at the genus or species level (Keren et al., 2020; Li et al., 2020).

To better understand the potential roles of nitrogen removal bacteria in membrane bioreactor (MBR) at high nitrite concentrations, we conducted a long-term nitrite inhibition experiment in MBR by progressively reducing the ammonium loading. Remarkably, an anammox self-recovery event was observed under nitrite inhibition, which has provided a potential solution to mitigate the inhibition of AnAOB in its industrial applications. Both 16S rRNA gene sequencing and genome-resolved metagenomic analysis were implemented to decode the shift of community composition and nitrogen metabolism genes during the nitrite-inhibition and recovery processes in the anammox system. Our finding will enable more stable control of anammox technology and facilitate its widespread application in wastewater treatment plants.

## Materials and Methods

### Membrane Bioreactor System

A continuous and complete-mix MBR system (Supplementary Figure 1) with an effective volume of 1.5 L and a 10-cm internal diameter was used in this study. The MBR was completely covered with aluminum foil to protect it from light. A hollow fiber ultrafiltration membrane (polyvinylidene fluoride) module was used to filter effluent and retain biomass through pumping. The MBR was mixed using a mechanical stirrer at 150 rpm to achieve a complete mix. A mixture of 95% argon gas and 5% carbon dioxide (50 ml min^–1^) was fed continuously into the MBR through a steel pipe extending to the bottom of the reactor to eliminate dissolved oxygen and maintain a circumneutral pH range of 6.8–8.0. Dissolved oxygen was monitored by a dissolved oxygen probe in the MBR and controlled at less than 1% air saturation. The entire system was maintained in a 37°C water bath. Mixed liquor volatile suspended solids (MLVSS) mass (0.9 g) of seed anammox sludge was sourced from a laboratory-scale anammox MBR; these anammox sludges were inoculated from an expanded granular sludge bed with carriers initially (Wu et al., 2019). The condition of this laboratory-scale anammox MBR was described as follows: the influent nitrite concentration of laboratory-scale anammox MBR was 1,550 mg N L^–1^, and the ammonia nitrogen concentration was 1,350 mg N L^–1^; the corresponding nitrogen removal rate was 1.5 g N L^–1^ day^–1^; MLVSS was approximately 3 g/L; the main AnAOB was *Ca.* Brocadia with a relative abundance of 19%. A synthetic nitrogenous wastewater medium (Supplementary Table 1) was used to cultivate AnAOB. A 24-h hydraulic retention time was maintained throughout the experiment. Nitrite and ammonium were added as needed in the forms of NaNO_2_ and NH_4_Cl. Before the commencement of the experiment, N_2_ was also sparged for 20 min into the medium to remove any dissolved oxygen.

### Long-Term Nitrite Inhibition

The anammox MBR was operated for a total of 280 days, which can be divided into two phases (Table 1). Phase I (days 1–110) encompassed the operation of the MBR in the acclimation (days 1–50) and stabilization (days 51–110) periods, whereas phase II (days 110–280) created the nitrite inhibition environment in the MBR. In phase I, AnAOB was acclimated from the seed sludge by continuous feeding of a simulated nitrogenous wastewater medium. The nitrogen loading rate was gradually increased once the nitrogen removal efficiency remained stable. The performance of the MBR was considered stabilized when the influent nitrite concentration reached 1,000 mg N L^–1^, and the effluent nitrite concentration was below 5 mg N L^–1^. Acclimation of AnAOB was continued for a further 60 days to maintain stability for subsequent experiments.

**TABLE 1:** Experimental phases during reactor maintaining.

Phase	Period	Days	NO_2_^–^-N inf	NH_4_^+^-N inf
			(mg L^–1^)	(mg L^–1^)
Phase I	Acclimation	1–51	20–1,000	20–950
	Stabilization	51–110	1,000	950
Phase II	Perturbation	110–127	1,000	950–930
	Inhibition	127–188	1,000	930–910
	Adaptation	188–228	1,000	910
	Recovery	228–280	1,000	910–870

**FIGURE 1:**
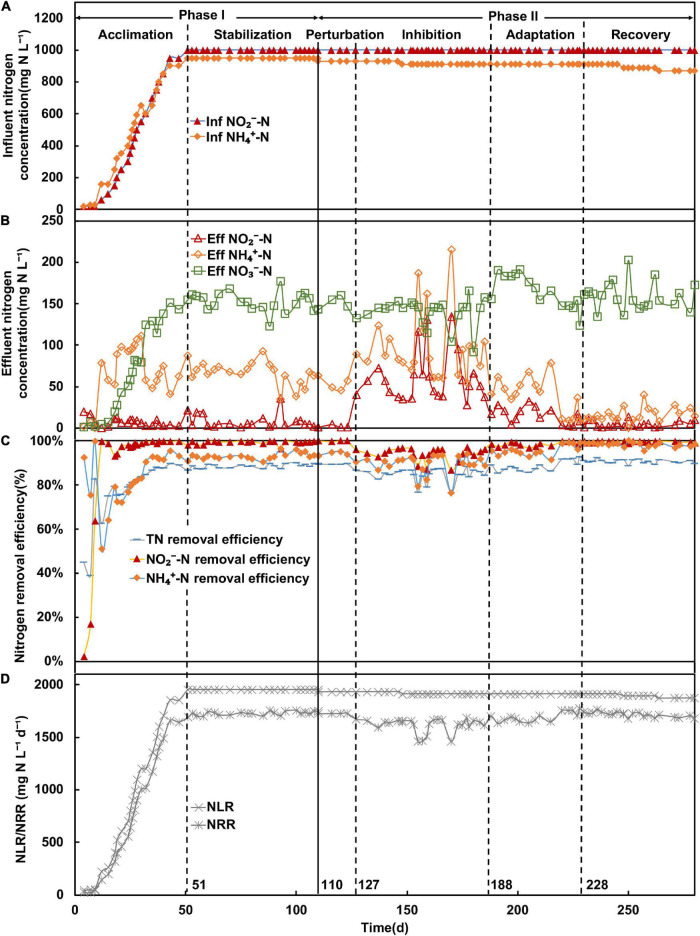
Performance of anammox membrane bioreactor; **(A)** concentration (mg N L^–1^) of influent ammonium (NH_4_^+^-N) and nitrite (NO_2_^–^-N); **(B)** concentration (mg N L^–1^) of effluent ammonium, nitrite, and nitrate (NO_3_^–^-N); **(C)** removal efficiency (%) of ammonium, nitrite, and total nitrogen (TN); **(D)** nitrogen removal rate (NRR) and nitrogen loading rate (NLR) (mg N L^–1^ day^–1^).

**FIGURE 2:**
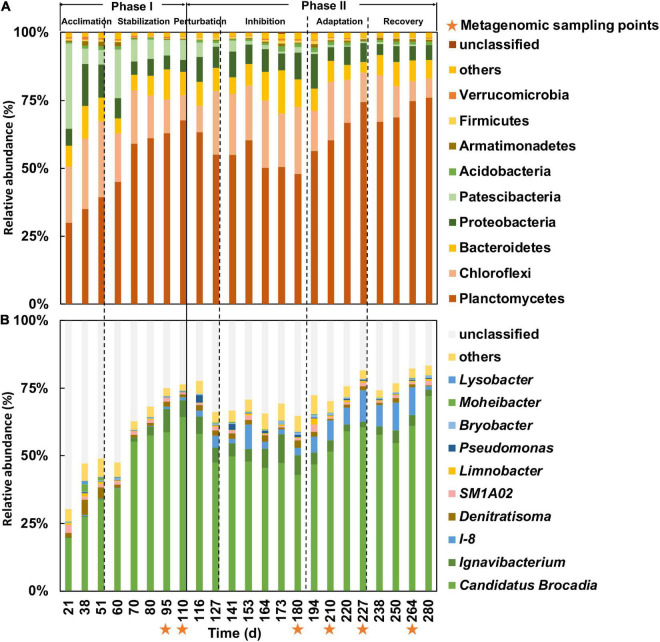
Composition of bacterial community at phylum **(A)** and genus **(B)** levels in samples over lifespan of bioreactor based on 16S rRNA OTUs. Orange stars below x-axis indicated sampling time points for metagenomics analysis.

Phase II was further subdivided into four periods based on the observed nitrogen removal efficiency, comprising of the perturbation (days 110–127), inhibition (days 127–188), adaptation (days 188–228), and recovery (days 228–280) periods. From days 110 to 127, the effluent nitrite and ammonium concentrations remained unchanged compared with the end of the stabilization period (Figure 1B), but the relative abundance of *Ca.* Brocadia decreased from 64 to 47% (Figure 2). Therefore, the system was considered disturbed, and days 110–127 were defined as the perturbation period. From the 127th day onward, the effluent nitrite and ammonium concentration (Figure 1B) increased rapidly along with the relative abundance of *Ca.* Brocadia further decreased to 43% (Figure 2B), suggesting the actual inhibition was happening. We, therefore, defined days 127–180 as the inhibition period. The nitrogen removal efficiency of days 188–228 began to rise, and the nitrogen removal efficiency of days 228–280 was completely restored (Figure 1C), so they were defined as the adaptation period and the recovery period, respectively.

Influent nitrite concentration was kept at 1,000 mg N L^–1^ throughout, whereas the influent ammonium concentration was gradually decreased from inhibition to recovery to create an imbalanced, nitrite-excess environment to inhibit AnAOB in phase II.

### Chemical Analyses

Influent and effluent samples were collected for chemical analyses and filtered through a 0.22-μm membrane filter (JIN TENG, China) two or three times a week. The concentrations of NO_2_^–^-N, NH_4_^+^-N, and NO_3_^–^-N were then measured using standard spectrophotometric methods (APHA, 2012). Four samples in three periods of MLVSS were determined using the standard methods (APHA, 2005): day 1 in the acclimation period, day 100 in the stabilization period, and days 150 and 180 in the recovery period.

### Sampling, DNA Extraction, and Sequencing

Sludge samples were collected at 23 different time points from MBR. Each time, CO_2_ was blown into the MBR to pump out the 50-ml mixed liquor before being collected in a 50-mL falcon tube. The collected sample was centrifuged at 4,000 rpm for 10 min to spin down the cell pellet. The supernatant was discarded, with the pellet aseptically transferred into a sterile 2-ml tube. All samples were stored at -80°C before DNA extraction. The DNA of 23 samples was extracted using the DNeasy Powersoil kit (QIAGEN, Germany) according to the manufacturer’s instructions. The DNA extracts were then subjected to barcoded polymerase chain reaction (PCR) amplification, with the barcodes added to the 5′ end of the PCR primers. The primers 515F (3′-GTGYCAGCMGCCGCGGTAA-5′) and 806R (5′- GGACTACNVGGGTWTCTAAT-3′) were used to amplify the V4 regions of bacterial 16S rRNA genes. PCR reaction solutions (50 μl) were prepared by mixing 25-μl 2 × Premix Taq (Takara Biotechnology, Dalian Co. Ltd., China), 1-μl forward and reverse primers (10 μM), and 3-μl DNA (20 ng/μl) template and nuclease-free water. Thermocycling conditions for PCR were set as follows: 5 min at 94°C for initialization; 30 cycles of 30-s denaturation at 94°C, 30-s annealing at 52°C, and 30-s extension at 72°C; followed by 10-min final elongation at 72°C. PCR products were purified with the TaKaRa MiNiBEST DNA Fragment Purification Kit Ver.4.0 (TaKaRa, Japan) and then quality-checked with gel electrophoresis. Sequencing libraries were generated using NEBNext ^®^ Ultra™ II DNA Library Prep Kit for Illumina ^®^ (New England Biolabs, MA, United States) following manufacturer’s recommendations, and index codes were added. The library quality was assessed on the Qubit@ 2.0 Fluorometer (Thermo Fisher Scientific, MA, United States). At last, the library was sequenced on an Illumina Nova6000 platform, and 250-bp paired-end reads were generated (Guangdong Magigene Biotechnology Co., Ltd., Guangzhou, China).

For shotgun metagenomic sequencing, a subset of six DNA samples were selected around six different time points in the four periods of the experiment: day 95 (D95) and day 110 (D110) in the stabilization period (days 51–110); day 180 (D180) in the inhibition period (days 127–188); day 210 (D210) and day 227 (D227) in the adaptation period (days 188–228); and day 264 (D264) from the recovery period (days 228–280). Each of the six samples is a mixture of the sludge from the days mentioned earlier and 3 days before and after. DNA was extracted and assessed before library preparation and sequenced on an Illumina NovaSeq 6000 platform generating 150-bp paired-end reads (Novogene Co., Ltd., Nanjing, China).

### 16S Ribosomal RNA Gene Analysis

The 16S rRNA gene sequence analysis was conducted using the Quantitative Insights Into Microbial Ecology (QIIME2) pipeline^1^ (Bolyen et al., 2019). DADA2 was used to filter low-quality sequences with lengths < 230 bp, remove chimeric sequences and singletons, and join the quality-filtered paired-end reads. Operational taxonomic units (OTUs) at ≥ 97% sequence similarity were generated by clustering sequences with the q2-vsearch method. After selecting a representative sequence for each OTU, sequences were aligned using mafft program, and the taxonomic identity was assigned using the Naive Bayes classifier with the SILVA 16S rRNA gene reference alignment database (release 123) (Pruesse et al., 2007) as the reference database. The taxonomic results were then generated at different levels for further analysis.

### Metagenomic Assembly and Binning

For shotgun metagenomic sequences, raw paired-end reads were initially filtered using fastp (Chen et al., 2018). Paired-end reads were filtered when the number of low-quality bases (*Q* ≤ 5) exceeded 50% in any sequence read. Filtered reads were assembled using MEGAHIT version 1.2.2 (Li et al., 2015), specifying a range of k-mer size values (79–149) in 20-bp intervals and finally reserved contigs > 1,000 bp.

The filtered contigs were processed with BASALT (Binning Across a Series of AssembLies Toolkit) (Yu et al., 2021) to obtain bins. The completeness and contamination of the bins were then estimated using CheckM version 1.0.13 (Parks et al., 2015) with lineage-specific marker genes and default parameters. Bins that meet the MIMAG standard (completeness- 5 * contamination ≥ 50%) (Parks et al., 2017) were retained in this study as metagenome-assembled genomes (MAGs) to infer potential nitrogen metabolism functionalities. Further details of MAGs were described in Supplementary File B.

### Taxonomic Assignment, Relative Abundance Calculations, and Functional Annotation of Metagenome-Assembled Genomes

One hundred twenty bacteria-specific protein sequences of conserved maker genes were identified from MAGs using GTDB-Tk version 0.3.2 (Chaumeil et al., 2019). Each protein sequence was individually aligned using hmmalign (Eddy, 2011) with default parameters. Individual alignments were concatenated and used as input to reconstruct the phylogenomic tree using the IQ-TREE version 1.6.12 (Nguyen et al., 2015) with the best-fit model of “LG + F + R10” and at 1,000 times bootstrapping (-b 1,000). Finally, the phylogenetic tree was visualized and edited in the iTOL version 4.4.2^2^ online platform (Letunic and Bork, 2019).

The relative abundance of MAGs was calculated by the average coverage multiplying the length of each MAG divided by the total read base pairs in each sample (Olm et al., 2017), as shown in Eq. (1). The detailed steps were provided in Supplementary File A.


(1) $$MAGabundance=\frac{coverage*lengthofMAG}{totalreadsofeachsample}$$

Genes were predicted from the MAGs using Prodigal version 2.6.2 (Hyatt et al., 2010), and predicted amino-acid sequences were annotated against the Kyoto Encyclopedia of Genes and Genomes database *via* the BLASTP program against the National Center for Biotechnology Information nr database with an *E*-value cutoff of 10^––5^. Nitrogen metabolic pathways were constructed using the Kyoto Encyclopedia of Genes and Genomes Mapper^3^ to visualize results.

## Results

### Membrane Bioreactor Performance

In the acclimation period, the acclimation of AnAOB was conducted by continuously increasing influent nitrite and ammonium concentrations (Figure 1A), with stable effluent nitrogen concentration observed (Figure 1B). The total nitrogen (TN) removal efficiency (Figure 1C) and nitrogen loading rate (Figure 1D) reached above 80% and 1,950 mg N^–1^ L^–1^ day^–1^ on the 25th day, whereas no significant fluctuation was observed after that, indicating the anammox system was successfully initiated. In this study, the system was maintained under stable conditions from the 51st day with NO_2_^–^-N, NH_4_^+^-N, and TN removal efficiencies at above 98, 91, and 87%, respectively. The MLVSS increased from 0.6 to 3.2338 g/L from inoculation to day 100. In the stabilization period, the average ratio of consumed nitrite over consumed ammonium (R_S_) and the ratio of produced nitrate over consumed ammonium (R_P_) values were 1.123 ± 0.021 and 0.172 ± 0.012, respectively.

In the perturbation period (days 110–127), no nitrite accumulation (Figure 1B) was observed, and the TN removal efficiency remained steady. However, in the inhibition period (days 127–188), the effluent NO_2_^–^-N concentration increased to 134 mg N L^–1^, whereas influent NH_4_^+^-N concentration continued to decrease (Figure 1A). The nitrogen removal efficiency was also observed to decline with fluctuations of both NH_4_^+^-N and NO_2_^–^-N removal efficiencies. In addition, the nitrogen removal rate declined from 1,670 to 1,458 mg N L^–1^ day^–1^, and the TN removal efficiency fluctuated within the range of 76–88% and decreased to a minimum of 76%. The NO_2_^–^-N and NH_4_^+^-N removal efficiencies were also unstable, whereas the NH_4_^+^-N removal efficiency generally decreased and reached a minimum of 76%. The MLVSS were 3.1857 and 3.1931 g/L on the 150th and 180th day in the inhibition period, respectively. In the recovery period, the influent ammonium concentration continued to decrease when the nitrite concentration remained steady (below 10 mg N L^–1^) (Figure 1B). The TN removal efficiency was recovered and maintained at approximately 90%, whereas NH_4_^+^-N and NO_2_^–^-N removal efficiency reached 96 and 99%, respectively, showing that the performance of MBR was restored.

### Characteristic of Microbial Assemblages in the Membrane Bioreactor System

A total of 3,855 OTUs were obtained from the 16S rRNA gene metabarcoding profiling of the 23 MBR samples across 280 days. A total of nine bacterial phyla with an abundance higher than 1% were identified in the samples (Figure 2A). Planctomycetes were found to be the predominant bacterial phylum of the communities in phase I, although their relative abundance fluctuated from 30 to 76% among the samples. After Planctomycetes, the main phyla in descending order of relative abundance are Chloroflexi, Bacteroidetes, and Proteobacteria. Chloroflexi, Bacteroidetes, and Proteobacteria have often been identified alongside Planctomycetes in other bioreactor studies (Lawson et al., 2017; Yang et al., 2018; Keren et al., 2020), which usually contain DN and DNRA bacteria (Guo et al., 2016; Zhao et al., 2019; Pan et al., 2020). The relative abundance of Planctomycetes reached 68% after acclimation in phase I (Figure 2A), which contained a large proportion of AnAOB (Figure 2B). In phase II, the relative abundance of Planctomycetes declined rapidly to 48% in the nitrite inhibition period before bouncing back to 76% in the recovery period. The relative abundance of Chloroflexi, however, increased to a maximum of 25% in the inhibition period and decreased to 7% in the recovery period.

A total number of 215 genera were identified in the samples. *Ca.* Brocadia, categorized as an AnAOB, was the dominant genus throughout the entire experiment (Figure 2B), whereas their relative abundance increased from 19 to 64% in phase I in concert with high nitrogen removal efficiency in the reactor. These results indicate that AnAOB could properly consume nitrite and ammonium in this phase. On the 180th day of the experiment, the relative abundance of *Ca.* Brocadia decreased to 43% in concert with high effluent nitrite concentration but then recovered to 72% in the later periods, indicating a disruption occurred due to the high nitrite concentration in the MBR. Apart from *Ca.* Brocadia, two genera, *Ignavibacterium* and *I-8*, were also observed with a combined high relative abundance of 11 and 12% in the inhibition and adaptation periods, respectively.

### Nitrogen Cycling Genes in the Metagenomic-Assembled Genomes Suggesting Nitrogen Removal Potentially Functioned in the Membrane Bioreactor

**FIGURE 3:**
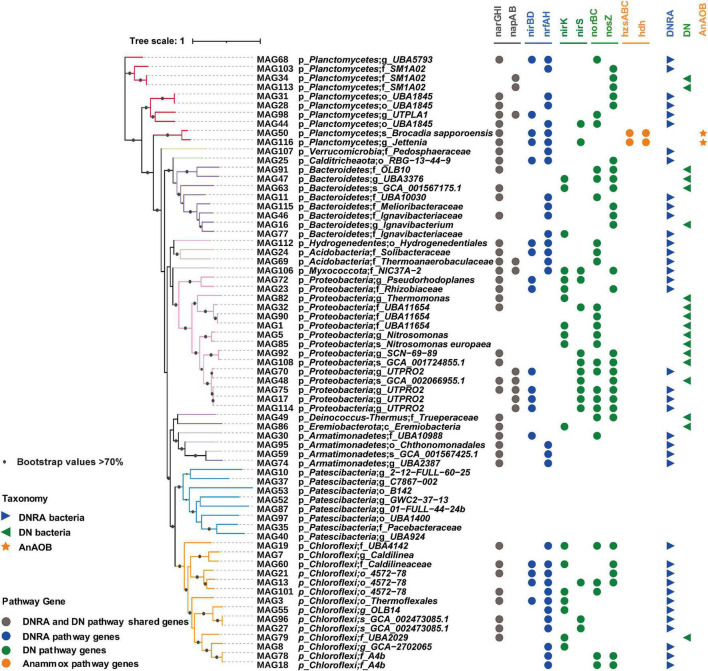
Phylogenomic reconstruction of high-quality MAGs from anaerobic ammonium oxidation (anammox) bioreactor communities. A phylogenetic tree was generated by maximum likelihood (ML) (inferred with IQ-tree and LG + F + R6 model) with 1,000 bootstrap replications. Different colored tree branches indicated different phylum levels. Bootstrap values > 70% were highlighted with black circles. Genes in various metabolic pathways were marked with different colored circles by iTOL version 4.4.2. Consequently, pathway of high-quality MAGs was summarized as follows: AnAOB (orange stars)—MAGs found with anammox genes (*hzsABC*/*hdh*), dissimilatory nitrate reduction (DNRA) bacteria (blue triangles pointing right)—MAGs found with DNRA genes (*nirBD*/*nrfAH*), and denitrifying only (DN) bacteria (green triangles pointing left)—MAGs only with DN genes (*nirS/nirK/norBC*/*nosZ*).

To precisely link microbial abundances to potential functions involved in nitrogen cycling, a metagenomic analysis was conducted to characterize the microbial functions in the whole reactor performance period. A total of 116 bins were recovered with metagenomic binning from six samples, of which 67 bins met the MIMAG standard (completeness - 5 * contamination ≥ 50% MAGs) and were considered as MAGs (Parks et al., 2017). These 67 MAGs were further analyzed and taxonomically classified based on phylogenetic analysis by taxonomic marker genes (Figure 3). The metabolic inference of these high-quality MAGs was then conducted to determine the nitrogen metabolic potential of the bacterial communities. A total of 58 MAGs across 12 phyla were involved in the nitrogen cycling activities. Two MAGs were taxonomically classified as *Ca. Brocadia sapporoensis* and *Ca. Jettenia*, respectively, with key functional genes for the anammox pathway observed, including hydrazine synthase (*hzsABC*) and hydrazine dehydrogenase (*hdh*), which reduce ammonium to hydrazine (N_2_H_4_) first and then N_2_. Therefore, the two MAGs were considered to represent AnAOB in the MBR. In addition to the AnAOB, 52 of 58 MAGs were found with potential DN functions, such as those with genes related to the first step of nitrite reduction (*nirS/nirK*), the second step of nitric oxide reduction (*norBC*), and the third step of nitrous oxide reduction (*nosZ*). Among these 52 MAGs, 30 MAGs with *nirS* or *nirK* genes were considered to be potentially involved in the nitrite reduction process. For the DNRA process, 38 of the 58 MAGs were found with DNRA functional genes (*nirBD* and *nrfAH*), which can potentially reduce nitrite to ammonium (Figure 3). Notably, 18 MAGs with potentials can only conduct the DN pathway (Figure 3).

### Dynamics of Anaerobic Ammonia-Oxidizing Bacteria, Denitrifying, and Dissimilatory Nitrate Reduction to Ammonium Bacteria in the Inhibition and Recovery Event

**FIGURE 4:**
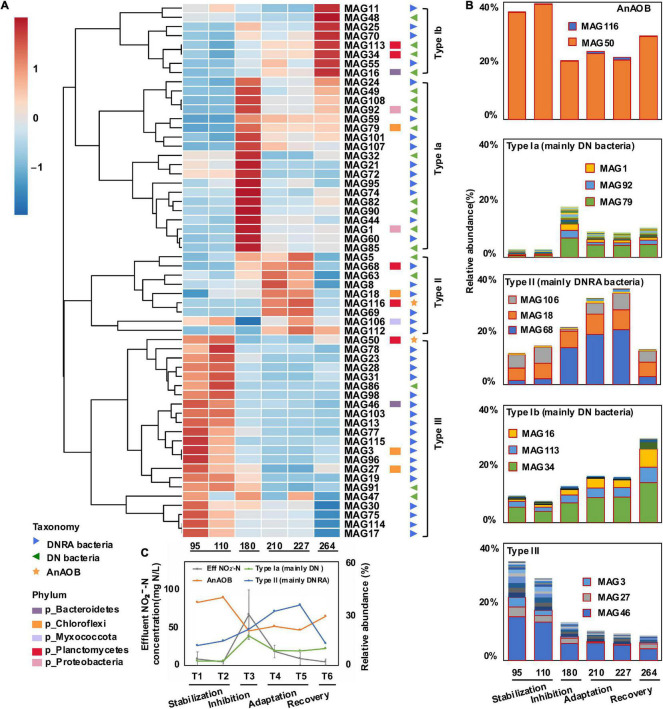
Analysis of bioreactor’s community clustering based on relative abundance of MAGs. A clustering heatmap of anammox (AnAOB), denitrifying (DN) bacteria, and dissimilatory nitrate reduction to ammonium (DNRA)-related metagenome-assembled genomes (MAGs) among experimental phases was generated at six time points **(A)**. Rows were first normalized, and heatmap was created based on a Euclidean distance matrix and clustered with complete method. Color scale was marked with high positive correlation in red and negative correlation in blue. Relative abundance of AnAOB and type Ia, Ib, II, and III bacteria were shown in **(B)**. MAGs with high relative abundance were marked in red boxes. Relative abundance of AnAOB, type Ib and type II bacteria at six time points (day 95, day 110, day 180, day 210, day 227, and day 264) as well as average effluent NO_2_^–^-N around sample collection time (T1: days 51–95, T2: days 96–110, T3: days 150–180, T4: days 188–210, T5: days 211–227, and T6: day 228–264) were shown in **(C)**.

To assess the variation of AnAOB, DN, and DNRA throughout the entire experiment, the relative abundances of MAGs were estimated based on metagenomic reads calculations. Among all MAGs, MAG50 and MAG116 were found with AnAOB-related genes, considered representative AnAOB genomes. Although genes required for the DNRA pathway were also found in the two MAGs, the high ammonium removal rate caused by the anammox process makes it safe to assume that the MAG50 and MAG116 bacteria mainly performed the anammox pathway to remove nitrogen (Keren et al., 2020). The relative abundance of MAG116 was less than 1%; therefore, the analysis was focused on MAG50. When the influent ammonium was decreased (Figure 1A), a clear pattern of increasing nitrite and ammonium concentrations was observed (Figure 1B), along with a decreasing relative abundance of MAG50 (*Ca.* Brocadia sapporoensis) from 39 to 20% (Figure 4C), suggesting the inhibition of the MAG50 species took place. In the later adaptation and recovery periods, we observed a decreasing nitrite concentration (Figure 1B), coupled with the relative abundance of MAG50 bouncing back up to 28% (Figure 4B) and nitrogen removal rate restored, implying that the inhibition effect was mitigated and the performance of the MAG50 species may have recovered along with its potential functions.

To further unfold how AnAOB was recovered and how DN/DNRA bacteria performed in these periods, a correlation analysis that linked nitrogen cycle genes with relative abundances of MAGs was conducted. Based on the dynamics of bacterial populations throughout the four periods, representative MAGs were clustered into three groups: type I (mainly DN bacteria), type II (mainly DNRA bacteria), and type III bacteria (mainly DNRA bacteria) (Figure 4B). Type I bacteria were further separated into two subgroups, types Ia and Ib, based on their rapid increasing time in the inhibition and recovery periods, respectively (Figures 4A,B). The MAG79 (family UBA2029 from Chloroflexi), MAG92 (genus *SCN-69-89* from Proteobacteria), and MAG1 (family *UBA11654* from Proteobacteria) were found as the three most abundant type Ia MAGs, whereas the MAG 34 (family SM1A02 from phylum Planctomycetes), MAG113 (family SM1A02 from phylum Planctomycetes), and MAG16 (genus *Ignavibacterium* from Bacterioidetes) were those of type Ib MAGs. Notably, all these three MAGs in the type I group affiliate to DN bacteria. The relative abundance of type II bacteria increased mainly in the adaptation period, including MAG68 (genus *UBA5793* from Planctomycetes), MAG18 (family *A4B* from Chloroflexi), and MAG106 (family *NIC37A-2* from Myxococcota) affiliating to DNRA bacteria. Type III bacteria showed a continuous relative abundance decrease from the inhibition to adaptation, containing MAG46 (family *Ignavibacteriaceae* from Bacteroidetes), MAG27 (*GCA_002473085.1* from Chloroflexi), and MAG3 (order Thermoflexales from Chloroflexi) affiliating to DNRA bacteria.

In the inhibition period, the relative abundance of type Ia bacteria increased rapidly from 3 to 17% (Figure 4C), whereas that of other bacterial groups either increased slowly (e.g., type Ib and II bacteria) or decreased (e.g., type III bacteria). In the adaptation period, the relative abundances of AnAOB were 22 and 20% on 210th and 227th day, respectively. The relative abundance of type II bacteria showed a major increase from 20 to 35%, whereas that of type Ia bacteria decreased from 17 to 8% along with a slow increase of that from 12 to 15% for type Ib bacteria. In the recovery period, the relative abundance of type II bacteria dropped from 35 to 13%, the same level as in the stabilization period, whereas that of type Ib bacteria had a rapid increase from 15 to 29% (Figure 4B). The relative abundance of type III bacteria, however, decreased steadily from 34 to 8% (Figure 4B).

## Discussion

In this study, we quickly established a laboratory-scale anammox-based nitrogen removal process using the MBR. Overall, a high and stable nitrogen removal rate had been achieved in the MBR after 51 days of operation and lasted in the stabilization period, indicating that high AnAOB activity had been achieved in the MBR. This coincided with the increase and stabilization of the relative abundance of Planctomycetes, especially the genus *Ca.* Brocadia. In the inhibition period, the low nitrogen removal efficiency and decreased relative abundance of *Ca.* Brocadia strongly suggested a disrupted anammox system, which is likely attributed to inhibiting AnAOB by a high nitrite concentration (Li et al., 2019; Lin et al., 2020; Pradhan et al., 2020). The concentration of the MLVSS has no significant change between the stabilization and inhibition periods, suggesting that the microbial biomass was maintained at a stable level. Therefore, we confer that the relative abundance of the microbial community at different time points was comparable. At the phylum level, the relative abundance of Chloroflexi increased, which is highly reasonable. Chloroflexi bacteria are highly active in protein degradation, which caters to the metabolism of soluble microbial products and extracellular polymeric substances derived from autotrophic organisms such as AnAOB in the MBR (Kindaichi et al., 2012). Besides, Chloroflexi bacteria play an essential role in enhancing the particle structure of the sludge by producing filamentous biomass networks on the sludge flocs and granules (Kragelund et al., 2011; Guo and Zhang, 2012; Kindaichi et al., 2012). At the genus level, *Ignavibacterium* was the most abundant except *Ca.* Brocadia, whose relative abundance variation was also related to the nitrite concentration change. Some *Ignavibacterium* spp. were previously reported contributing to the nitrogen removal through the nitrite or nitrate pathway (Yang et al., 2017), whereas some other *Ignavibacterium* spp. were capable of heterotrophic denitrification (Liu et al., 2012; Oren, 2014). In the recovery period, we may imply the recovery of AnAOB by the restoration of nitrogen removal efficiency (Zekker et al., 2015; Zhang et al., 2016). It is critical to explore such a recovery process because it may directly or indirectly be linked with certain bacteria such as DNRA and DN, which can consume the excess nitrite in the inhibition and recovery period. Therefore, we divided these MAGs into three groups: type I separating into two subgroups: types Ia and Ib (mainly DN bacteria), type II (mainly DNRA bacteria), and type III bacteria (mainly DNRA bacteria) (Figure 4B).

Based on these three groups, we further analyzed the dynamics of microbial population in the event of anammox inhibition and restoration to dissect the potential functions of nitrogen cycling bacteria. The results show the relative abundance of type Ia bacteria increased rapidly along with nitrite concentration, whereas other bacterial groups increased slowly or decreased in the inhibition period. We infer that type Ia bacteria are better adapted to the high nitrite concentration (60.55 ± 29.20 mg N L^–1^). The nitrogen removal efficiency increased from a minimum of 76–89% from days 170 to 188 in the inhibition period (Figure 1C), indicating excess nitrite was consumed. Therefore, type Ia bacteria may serve as pioneers to consume the excess nitrite. Such speculation is supported by a previous study, which revealed DN bacteria from Proteobacteria could aid in reducing nitrite to nitrogen gas. Based on the metagenomic analysis, the nitrite denitrification genes in DN bacteria had higher abundance after the inhibition phase, and their cooperation could prevent the nitrite inhibition of anammox bacteria when the influent nitrite concentration was higher (Zhao et al., 2019). Therefore, we assume that type Ia DN bacteria are important nitrite scavengers at high nitrite concentrations.

In the adaptation period, nitrite concentration rapidly dropped (15.33 ± 9.52 mg N L^–1^), whereas the relative abundance of AnAOB did not fluctuate significantly, suggesting that the anammox bacteria may not recover under this nitrite concentration. On the other hand, we observed a major increase of type II bacteria, a decrease of type Ia bacteria, and a slow rise in type Ib. This shows that type I bacteria (mainly DN bacteria) were outcompeted by type II bacteria in this period. The competition mechanisms between the two under high nitrite concentration conditions remain to be revealed in future studies. More importantly, nitrite and ammonium concentrations decreased in the adaptation period compared with inhibition. The effluent nitrite concentration maintained at 19 ± 8 mg N L^–1^, whereas the ammonium concentration was observed with an increase from 41 to 78 mg N L^–1^ before day 215 in the adaptation period (Figure 1B) (effluent NH_4_^+^-N/NO_2_-N ratio increased from 2.48 to 3.66). As DNRA can provide NH_4_^+^ for the anammox process in NH_4_^+^-limiting environments (Wang et al., 2018), the ammonium accumulation might be attributed to the DNRA process, which might be critical in triggering the recovery of AnAOB. Therefore, apart from type Ia bacteria that reduce nitrite concentration, type II bacteria might be another key player in assisting the AnAOB recovery.

In the recovery period, the nitrite concentration decreased to a low level of 4.63 ± 3.66 mg N L^–1^ (Figure 4C), whereas AnAOB and nitrogen removal efficiency (Figure 1C) were observed to have gradually recovered and stabilized. The relative abundance of type II bacteria again dropped to the same level as in the stabilization period. On the other hand, the relative abundance of type III bacteria decreased steadily, and type Ib bacteria increased rapidly, suggesting that type III DNRA bacteria (Figure 4B) were irreversibly stressed under high nitrite concentration (Figure 4C) and replaced by type Ib bacteria. The observations discussed earlier imply that the anammox performance was restored along with the shift of the microbial community. In the restored anammox system, although the relative abundance of type II bacteria remained similar to that under the initial conditions, type III bacteria were substituted by type I bacteria (mostly type Ib). It is indicated that different niches of bacteria may occur after inhibition in the MBR system, which contributes to the stability of the micro-ecosystem.

## Conclusion

In this study, a laboratory-scale anammox bioreactor was used to conduct an investigation of the microbial shift and functional interactions of AnAOB, DN, and DNRA bacteria during a long-term nitrite inhibition to eventual self-recovery episode. The relative abundance of AnAOB first decreased due to high nitrite concentration, which lowered the system’s nitrogen removal efficiency but then recovered automatically without any external interference. By analyzing the relationship between the relative abundances of MAGs and their nitrogen metabolism pathways, we found that type Ia (mainly DN) and type II (mainly DNRA) bacteria outcompeted other bacteria in the inhibition and adaptation periods, respectively. They were capable of living under high nitrite concentration conditions and potentially served as a nitrite scavenger that could trigger the recovery of AnAOB from the nitrite inhibition. Our results provide a possible mechanistic explanation for the performance shift of the anammox bioreactor during a long-term nitrite inhibition to the eventual self-recovery episode and advance the stable control of this promising technology.

## Data Availability Statement

The datasets presented in this study can be found in online repositories. The names of the repository/repositories and accession number(s) can be found below: https://www.ncbi.nlm.nih.gov/, PRJNA608637.

## Author Contributions

XQ: conceptualization, methodology, experimental operation, software, investigation, data analysis, writing—original draft, and visualization. LZha: methodology, experimental operation, software, investigation, and data analysis. ZQ and LW: methodology, data analysis, and revision. YaW and YuW: bioreactor maintenance and operation. CD: methodology and software. JS: methodology. XZ: experimental operation. BL, LZho, AM, and W-QZ: revision. KY: supervision, funding acquisition, resources, and revision. All authors contributed to the article and approved the submitted version.

## Conflict of Interest

The authors declare that the research was conducted in the absence of any commercial or financial relationships that could be construed as a potential conflict of interest.

## Publisher’s Note

All claims expressed in this article are solely those of the authors and do not necessarily represent those of their affiliated organizations, or those of the publisher, the editors and the reviewers. Any product that may be evaluated in this article, or claim that may be made by its manufacturer, is not guaranteed or endorsed by the publisher.

## Footnotes

1
https://qiime2.org

2
https://itol.embl.de/personal_page.cgi

3
http://www.genome.jp/kegg/tool/map_pathway.html
