# Supplementary material for: Specific Denitrifying and Dissimilatory Nitrate Reduction to Ammonium Bacteria Assisted the Recovery of Anammox Community From Nitrite Inhibition
Source: Front Microbiol. 2022 Jan 20;12:781156. doi: 10.3389/fmicb.2021.781156 (PMC8811301; doi:10.3389/fmicb.2021.781156)
Supplement: Supplementary file 1 [file Data_Sheet_1.zip › Supplementary Material/Supplementary File A.docx]

**Supplementary Material A**

**16S rRNA gene analysis**

DADA2 was used to filter low-quality sequences with lengths < 230 bp, remove chimeric sequences, remove singletons, and join the quality-filtered paired-end reads. Operational taxonomic units (OTUs) at ≥ 97% sequence similarity were generated by clustering sequences with the q2-vsearch method. After selecting a representative sequence for each OTU, sequences were aligned using mafft program and the taxonomic identity was assigned using Naive Bayes classifier with the SILVA 16S rRNA gene reference alignment database (release 123) (Pruesse et al., 2007) as reference database.

**The relative abundances of the MAGs**

The relative abundances of the MAGs were calculated by several steps. The MAGs were firstly combined as a reference file and bowtie2 (Langmead&Salzberg, 2012) was used to construct a comparison template, that is, to establish an index. Next, each set of raw data was mapped to the reference file, then a customized script was used for coverage and length calculations (<http://github.com/banfieldlab/mattolm-public-scripts>).

**Table S1. Composition of (A) medium, (B) trace element solution I and (C) trace element solution II used in MBR system**

**(A)** Medium composition

| Composition | Concentration（g·L^-1^） |
| --- | --- |
| NaHCO_3_ | 1.25 |
| KH_2_PO_4_ | 0.01 |
| CaCl_2_·2H_2_O | 0.015 |
| MgCl_2_·6H_2_O | 0.0813 |
| FeSO_4_·7H_2_O | 0.01379 |
| EDTA | 0.00625 |
| Trace element solution I | 1mL |
| Trace element solution II | 1mL |
| NaNO_2_ | Make for need |
| NH_4_Cl | Make for need |
|  |  |

**(B)** Trace element solution I composition

| Composition | Concentration（g·L^-1^） |
| --- | --- |
| ZnSO_4_·7H_2_O | 0.43 |
| CoCl_2_·6H_2_0 | 0.24 |
| MnCl_2_·4H_2_O | 1.0 |
| CuSO_4_·H_2_O | 0.25 |
| NaMoO_4_·2H_2_O | 0.22 |
| NiCl_2_·6H_2_O | 0.19 |
| H_3_BO_4_ | 0.014 |

**(C)** Trace element solution II composition

| composition | concentration（g·L^-1^） |
| --- | --- |
| NaSeO_4_·10H_2_O | 0.21 |
| NaWO_4_·2H_2_O | 0.05 |

**Table S2.** Statistics for separate assembly and co-assembly of the metagenomics samples.

| Samples | Number contigs | assembly sizes(bp) | N50(bp) | Mean contig length (bp) |
| --- | --- | --- | --- | --- |
| D66_ assembly | 34,736 | 173,885,969 | 17,702 | 5,006 |
| D109_ assembly | 45,510 | 187,841,850 | 99,46 | 4,127 |
| D279_ assembly | 18,335 | 102,591,006 | 28,167 | 5,595 |
| D283_ assembly | 20,771 | 111,795,520 | 25,966 | 5,382 |
| D445_ assembly | 33,083 | 158,987,254 | 11,787 | 4,806 |
| D527_ assembly | 37,006 | 167,406,033 | 12,252 | 4,524 |
| D537_ assembly | 26,007 | 121,550,016 | 16,326 | 4,674 |
| D546_ assembly | 24,092 | 118,800,986 | 17,827 | 4,931 |
| Co_assembly | 75,990 | 440,464,447 | 21,986 | 5,796 |

**Figure S1.** The structure of Membrane bioreactor (MBR) system.

**REFERENCES**

Langmead, B., and Salzberg, S. L. (2012). Fast gapped-read alignment with Bowtie 2. *Nat. Methods* 9(4), 357-359. doi:10.1038/nmeth.1923

Pruesse, E., Quast, C., Knittel, K., Fuchs, B. M., Ludwig, W., Peplies, J., et al. (2007). SILVA: a comprehensive online resource for quality checked and aligned ribosomal RNA sequence data compatible with ARB. *Nucleic Acids Res.* 35(21), 7188-7196. doi:10.1093/nar/gkm864
